# Zeaxanthin and Lutein Ameliorate Alzheimer’s Disease-like Pathology: Modulation of Insulin Resistance, Neuroinflammation, and Acetylcholinesterase Activity in an Amyloid-β Rat Model

**DOI:** 10.3390/ijms25189828

**Published:** 2024-09-11

**Authors:** Da-Sol Kim, Suna Kang, Na-Rang Moon, Bae-Keun Shin, Sunmin Park

**Affiliations:** 1 Department Food and Nutrition, Hoseo University, Asan 31499, Republic of Korea; tpfpttm14@daum.ent (D.-S.K.); roypower003@naver.com (S.K.); a213900@hanmail.net (N.-R.M.); swoed567@nate.com (B.-K.S.); 2 Department of Bioconvergence, Hoseo University, Asan 31499, Republic of Korea

**Keywords:** carotenoids, hippocampal insulin signaling, systemic insulin resistance, memory impairment

## Abstract

Alzheimer’s disease (AD) is characterized by impaired insulin/insulin-like growth factor-1 signaling in the hippocampus. Zeaxanthin and lutein, known for their antioxidant and anti-inflammatory properties, have been reported to protect against brain damage and cognitive decline. However, their mechanisms related to insulin signaling in AD remain unclear. This study investigated the efficacy and mechanisms of zeaxanthin, lutein, and resveratrol in modulating an AD-like pathology in an amyloid-β rat model. Rats were administered hippocampal infusions of 3.6 nmol/day amyloid-β (Aβ)(25-35) for 14 days to induce AD-like memory deficits (AD-CON). Normal control rats received Aβ(35-25) (Normal-CON). All rats had a high-fat diet. Daily, AD rats consumed 200 mg/kg body weight of zeaxanthin (AD-ZXT), lutein (AD-LTN), and resveratrol (AD-RVT; positive-control) or resistant dextrin as a placebo (AD-CON) for eight weeks. The AD-CON rats exhibited a higher Aβ deposition, attenuated hippocampal insulin signaling (reduced phosphorylation of protein kinase B [pAkt] and glycogen synthase kinase-3β [pGSK-3β]), increased neuroinflammation, elevated acetylcholinesterase activity, and memory deficits compared to the Normal-CON group. They also showed systemic insulin resistance and high hepatic glucose output. Zeaxanthin and lutein prevented memory impairment more effectively than the positive-control resveratrol by suppressing acetylcholinesterase activity, lipid peroxidation, and pro-inflammatory cytokines (*TNF-α*, *IL-1β*). They also potentiated hippocampal insulin signaling and increased brain-derived neurotrophic factor (*BDNF*) and ciliary neurotrophic factor (*CTNF*) mRNA expression to levels comparable to the Normal-CON rats. Additionally, zeaxanthin and lutein improved glucose disposal, reduced hepatic glucose output, and normalized insulin secretion patterns. In conclusion, zeaxanthin and lutein supplementation at doses equivalent to 1.5–2.0 g daily in humans may have practical implications for preventing or slowing human AD progression by reducing neuroinflammation and maintaining systemic and central glucose homeostasis, showing promise even when compared to the established neuroprotective compound resveratrol. However, further clinical trials are needed to evaluate their efficacy and safety in human populations.

## 1. Introduction

Alzheimer’s disease (AD) is a progressive neurodegenerative disorder characterized by the abnormal accumulation of amyloid-beta (Aβ) peptides, which form extracellular plaques, and tau protein hyperphosphorylation and aggregation in the form of intracellular neurofibrillary tangles [1]. It results in synaptic dysfunction, neuronal death, and cognitive impairment [2]. Although the precise etiology of AD remains unclear, oxidative stress, neuroinflammation, mitochondrial dysfunction, and insulin resistance have been implicated as the major factors contributing to AD pathogenesis [3,4,5].

Emerging evidence suggests impaired insulin signaling and resistance play crucial roles in AD pathology [6]. Insulin regulates tau phosphorylation, the critical step in the formation of neurofibrillary tangles, through the modulation of the kinases and phosphatases, and insulin signaling deficiency in the brain is believed to result in tau hyperphosphorylation [7]. Additionally, insulin resistance exacerbates Aβ accumulation and neuroinflammation, further driving AD pathogenesis [4,7]. However, few studies have examined the association between glucose metabolism and cognitive function.

Medications currently approved for AD provide only modest symptomatic relief without targeting the underlying disease mechanisms [8,9]. Moreover, many investigational drugs have failed clinical trials, highlighting the urgent need for alternative multi-targeted therapeutic strategies [10]. Early interventions may be more effective in delaying or preventing the onset of AD. Functional foods and nutraceuticals have gained significant interest due to their ability to modulate the pathways implicated in AD pathogenesis, including oxidative stress, neuroinflammation, insulin resistance, and neuronal dysfunction [5,11,12].

Zeaxanthin and lutein are dietary carotenoids with potent antioxidant and anti-inflammatory properties and are promising preventive agents for AD [13,14]. Resveratrol, a polyphenolic compound found in grapes and red wine, has demonstrated neuroprotective effects in preclinical studies by improving insulin sensitivity and cognitive function [15]. Previous research has shown the efficacy of these compounds in reducing the Aβ burden, attenuating oxidative damage, suppressing neuroinflammation, and improving cognition in AD animal models [13,16]. However, the precise mechanisms underlying their neuroprotection and effects on insulin resistance remain to be fully elucidated. Therefore, this study aimed to investigate the efficacy and explore the efficacy and action mechanisms of zeaxanthin and lutein in modulating an AD-like pathology and insulin secretion and resistance in an Aβ(25-35)-infused rat model. We hypothesized that supplementation with these natural compounds would mitigate Aβ deposition, insulin signaling, oxidative stress, and neuroinflammation in the hippocampus and enhance insulin resistance and secretion. Resveratrol was used as the positive control to compare the efficacy and mechanisms of the two compounds. Elucidating their multi-targeted neuroprotective mechanisms could provide insights into alternative preventive and therapeutic strategies for AD.

## 2. Results

### 2.1. Aβ Accumulation 

**Figure 1:**
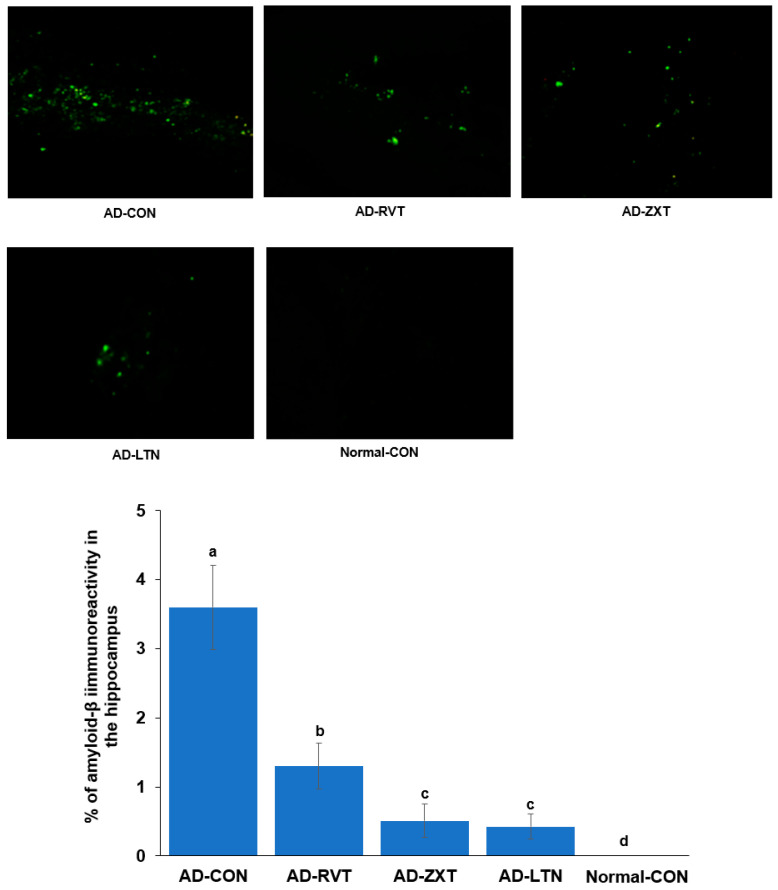
Amyloid-β accumulation in the hippocampus. Image amyloid-β deposition in the hippocampus (X100 magnification). Green dots are shown as amyloid-β deposition represented by immunohistochemistry with anti-amyloid-β antibody. The percentage of amyloid-β immunoreactivity in the hippocampus (n = 8 for each group) is shown. AD-CON (control), AD-LTN, AD-ZXT, and AD-RVT (positive-control) represent the groups of assigned 200 mg/kg bw/day resistant dextrin, luteolin, zeaxanthin, or resveratrol in amyloid-β-infused rats. Normal-Con rats infused with β-amyloid (35-25) received and consumed 200 mg/kg bw/day resistant dextrin. Different letters (a, b, c, d) on the bars are significantly different from each other (*p* < 0.05) by Tukey’s post hoc test. Bars with the same letter (a, a) are not significantly different.

The infusion of Aβ in the hippocampus resulted in the accumulation of Aβ as observed in the AD-CON group. The treatment with zeaxanthin and lutein resulted in a decrease in Aβ accumulation of 11.4% and 10.1% of the amount of the AD-CON, respectively, but not as much as the Normal-CON (*p* < 0.001). The level of Aβ accumulation in the AD-RVT group was 33.5% of the amount of the AD-CON, which was greater than that of AD-LTN and AD-ZXT (Figure 1). 

### 2.2. Memory Function

**Figure 2:**
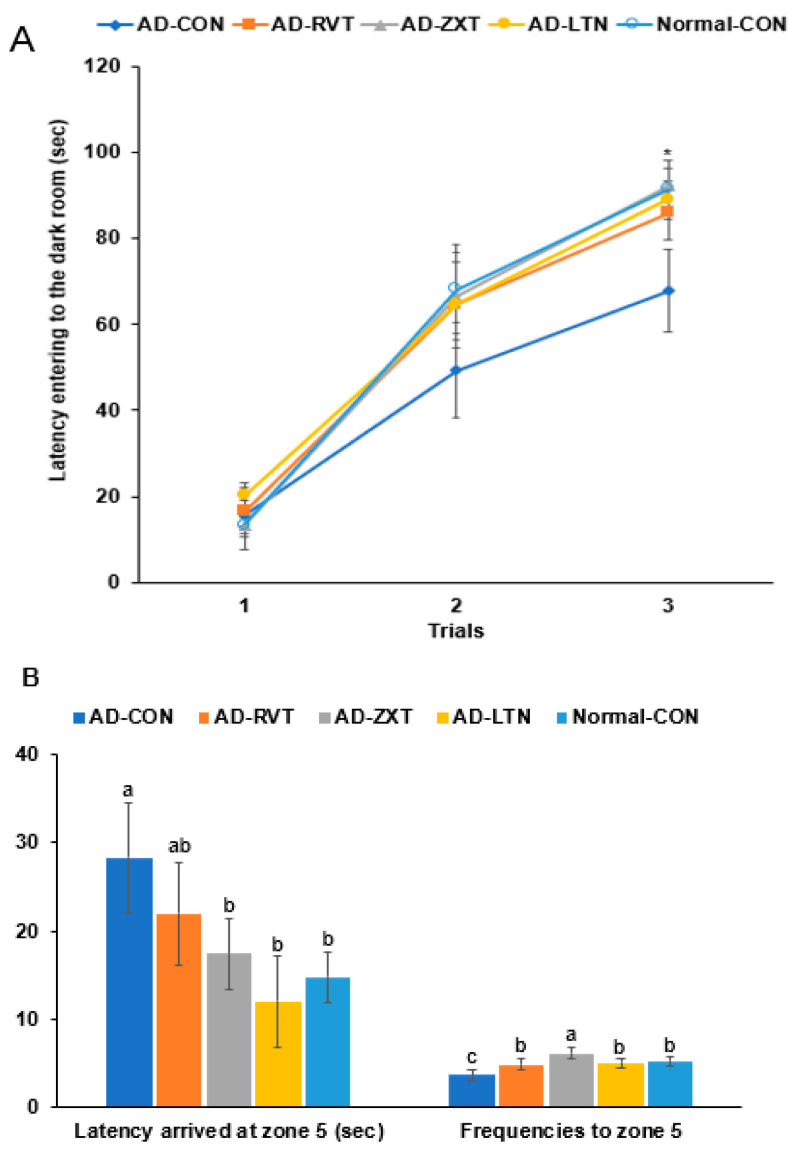
Memory function. (**A**). Latency time to enter the darkroom in the passive avoidance test (n = 16). (**B**). Latency time and frequencies of locating zone 5 located the platform on day 5 during the water maze test (n = 16). Each dot or bar with an error bar represents mean ± SD (n = 16 for each group). The β-amyloid (25-35)-infused rats were fed high-fat diets with 200 mg/kg bw/day resistant dextrin (AD-CON; control), lutein (AD-LTN), zeaxanthin (AD-ZXT), or resveratrol (AD-RVT; positive-control). The rats infused with β-amyloid (35-25) were fed a high-fat diet containing 200 mg/kg bw/day resistant dextrin, serving as normal controls (Normal-CON, Non-AD). * Significantly different at the time point among the groups at <0.05. Different letters (a, b, c) on the bars are significantly different from each other (*p* < 0.05) by Tukey’s post hoc test. Bars with the same letter (a, a) are not significantly different.

In the passive avoidance test, the latency to enter the dark room was shorter in the 2nd and 3rd trials in the AD-CON group than in the Normal-CON group. The AD-RVT, AD-ZXT, and AD-LTN groups showed increased latency, which was similar to the Normal-CON group (*p* < 0.05; Figure 2A). This suggests that short-term memory decreased in the AD-CON group, which improved with carotenoids and resveratrol treatment. 

In the water maze test, the latency to zone 5 at the 3rd trial was longer in the AD-CON than in the Normal-CON group. The latency was shorter by 56.4% in the AD-ZXT group and by 43.5% in the AD-LTN group than in the AD-CON group (*p* < 0.05). Their latency was similar to the Normal-CON, suggesting that the intake of zeaxanthin and lutein reduced the latency (Figure 2B). The latency was lower in the AD-RVT group than in the AD-CON group, suggesting that the resveratrol intake decreased the latency but not as much as the Normal-CON group. The frequencies of visiting zone 5, located on the podium, were less in the AD-CON than in the Normal-CON group, and they increased in the supplementation groups (*p* < 0.01). The AD-ZXT group showed higher frequencies than the Normal-CON group (Figure 2B). 

### 2.3. Hippocampal Neuroinflammation, BDNF and CNTF mRNA Expressions, and Insulin Signaling

The hippocampal triglyceride and cholesterol levels were higher in the AD-CON group compared to the Normal-CON group (Table 1). However, the levels of these lipids were lower in the AD-ZXT, AD-LTN, and AD-RVT groups than in the AD-CON group and similar to the Normal-CON group (*p* < 0.05), indicating that zeaxanthin, lutein, and resveratrol supplementation prevented this increase. Conversely, the hippocampal glycogen content was lower in the AD-CON group than in the Normal-CON group, and the levels were higher in the AD-ZXT, AD-LTN, and AD-RVT groups (*p* < 0.05), suggesting zeaxanthin, lutein, and resveratrol supplementation prevented this decrease (Table 1). The hippocampal lipid peroxide levels, a marker of oxidative stress, were higher in the AD-CON group than in the Normal-CON group. The levels were lower in the AD-RVT, AD-ZXT, and AD-LTN groups than the AD-CON (*p* < 0.01), suggesting resveratrol, zeaxanthin, and lutein supplementation effectively suppressed this increase (Table 1).

The hippocampal acetylcholinesterase (AChE) activity, a key enzyme linked to Alzheimer’s disease pathology, was higher in the AD-CON group compared to the Normal-CON group (Table 1). The AChE e activity was lower in the AD-RVT, AD-ZXT, and AD-LTN groups compared to the AD-CON group (*p* < 0.01). However, their levels of AChE activity were higher than that of the Normal-CON, suggesting that resveratrol, zeaxanthin, and lutein supplementation reduced the AChE activity, with zeaxanthin and lutein exhibiting a more potent suppression than resveratrol (Table 1).

The levels of pro-inflammatory cytokines, *TNF-α* and *IL-1β*, markers of neuroinflammation, and their mRNA expression in the hippocampus were significantly higher in the AD-CON group compared to the Normal-CON group (Table 1). Resveratrol, zeaxanthin, and lutein supplementation effectively suppressed the increase in these inflammatory markers, as observed in the AD-RVT, AD-ZXT, and AD-LTN groups (*p* < 0.05), with the AD-LTN group exhibiting values similar to the Normal-CON group (Table 1). Conversely, the mRNA expression of the neurotrophic factors, *BDNF* and *CNTF*, which are important for neuronal survival and function, was significantly higher in the Normal-CON group compared to the AD-CON group. The expression of *BDNF* and *CNTF* was higher in the AD-RVT, AD-ZXT, and AD-LTN groups compared to the AD-CON group (*p* < 0.01 and *p* < 0.01), indicating that resveratrol, zeaxanthin, and lutein supplementation increased the expression of these factors in the AD rats (Table 1).

**Figure 3:**
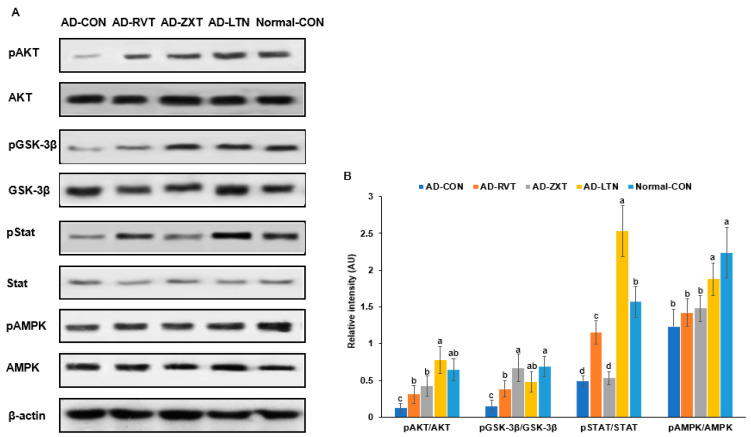
Hippocampal insulin signaling. (**A**). Immunoreactivity blots. (**B**). Intensity of the bands. After preparing the hippocampus lysates, the phosphorylation and expression of proteins related to insulin signaling were measured using a Western blot analysis, and their density was determined. Each dot and bar with error bars represent mean ± SD (n = 4 for each group). The amyloid-β (25-35)-infused rats were fed high-fat diets with 200 mg/kg bw resistant dextrin (AD-CON; control), lutein (AD-LTN), zeaxanthin (AD-ZXT), or resveratrol (AD-RVT; positive-control). The rats infused with amyloid-β (35-25) were fed a high-fat diet containing 200 mg resistant dextrin per kg bw and served as the normal control (Normal-CON, Non-AD). Different letters (a, b, c, d) on the bars are significantly different from each other (*p* < 0.05) by Tukey’s post hoc test. Bars with the same letter (a, a) are not significantly different. Akt, protein kinase A; GSK-3β, glycogen synthase kinase-3β; STAT-3, signal transducer and activator of transcription 3; AMPK, AMP kinase.

The intensity of phosphorylated Akt, a key mediator of insulin signaling, in the hippocampus, was lower in the AD-CON group compared to the Normal-CON group (Figure 3). The phosphorylation of Akt in the AD-ZXT and AD-LTN groups was higher compared to the AD-CON group and similar to the Normal-CON group. It was higher in the AD-RVT group, though not as high as in the AD-ZXT and AD-LTN groups. (*p* < 0.001; Figure 3). This suggests that zeaxanthin and lutein supplementation increased Akt phosphorylation, similarly to what is observed in normal rats. While resveratrol also elevated Akt phosphorylation, the increase was lower than that due to zeaxanthin and lutein. The phosphorylation of glycogen synthase kinase-3 beta (GSK-3β), a downstream target of Akt (*p* < 0.01), followed a pattern similar to that of Akt phosphorylation. The phosphorylation of the signal transducer and activator of transcription 3 (STAT3), another insulin signaling mediator, was lower in the AD-CON group than in the Normal-CON group. Compared to the AD-CON group, the phosphorylation of STAT3 was higher in the AD-RVT and AD-LTN groups but not in the AD-ZXT group (*p* < 0.001; Figure 3). The phosphorylation of AMPK, an energy sensor, was higher in the AD-LTN than AD-CON group, and it was similar to the Normal-CON (*p* < 0.05; Figure 3). 

### 2.4. Energy Metabolism

The final body weight and weight gain during the experimental period were lower in the AD-CON group than in the Normal-CON group, while it increased in the AD-ZXT and AD-LTN groups compared to the AD-CON group (*p* < 0.05 and *p* < 0.05). The AD-RVT did not prevent the decrease in weight gain. However, the weight gain by the lutein and zeaxanthin intake during intervention was not as much as in the Normal-CON group (Table 2). Therefore, weight changes were partly linked to AD status and the supplementations affected weight gain by improving AD status. However, the visceral fat content was not significantly different among the groups. Despite having a similar visceral fat mass, the serum concentrations of *TNF-α* were much higher in the AD-CON group compared to the Normal-CON group and lower in the AD-RVT, AD-ZXT, and AD-LTN groups (*p* < 0.05), indicating that resveratrol, zeaxanthin, and lutein supplementation suppressed its increment (Table 2). 

### 2.5. Glucose Metabolism

**Figure 4:**
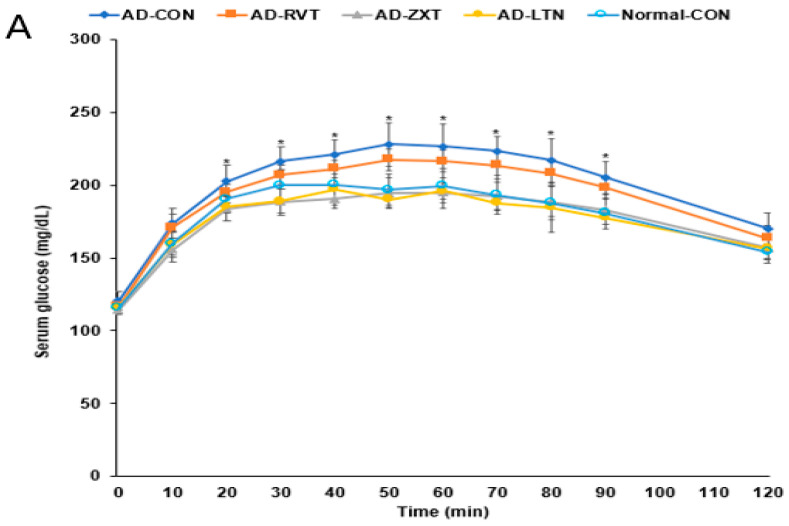

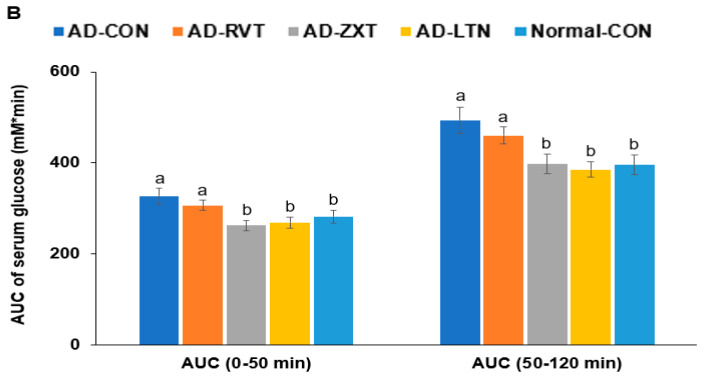
Oral glucose tolerance test with oral glucose intake (2 g/kg body weight). (**A**). Changes in the serum glucose levels (n = 16). (**B**). Area under the curve (AUC) of glucose. Each dot and bar with error bars represent (n = 16). Mean ± SD (n = 16 for each group). The β-amyloid (25-35)-infused rats were fed high-fat diets with 200 mg/kg bw resistant dextrin (AD-CON; control), lutein (AD-LTN), zeaxanthin (AD-ZXT), or resveratrol (AD-RVT; positive-control). The rats infused with β-amyloid (35-25) were fed a high-fat diet containing 200 mg resistant dextrin per kg bw and served as the normal control (Normal-CON, Non-AD). * Significantly different at the time point among the groups at <0.05. Different letters (a, b) on the bars are significantly different from each other (*p* < 0.05) by Tukey’s post hoc test. Bars with the same letter (a, a) are not significantly different.

There were no significant differences in the fasting serum glucose concentrations among the groups. However, fasting serum insulin concentrations were higher in the AD-CON group than in the Normal-CON group (Table 2). HOMA-IR exhibited an insulin resistance much higher in the AD-CON group than in the Normal-CON group, decreasing in the order of AD-CON, AD-RVT, AD-ZXT, and AD-LTN = Normal-CON (*p* < 0.001). After the administration of 2 g glucose per kg bw, the serum glucose concentrations increased up to 60 min in the AD-CON group, but it was elevated up to 30 min in the Normal-CON group. In the AD-RVT, AD-ZXT, and AD-LTN groups, the glucose concentrations increased up to 50 min (Figure 4A). The peak serum glucose concentrations were much higher in the AD-CON group than in the Normal-CON group. In the AD-ZXT and AD-LTN groups, the peak decreased and was similar to the Normal-CON group (*p* < 0.05; Figure 4A). The changes in serum glucose concentrations suggested that compared to the Normal-CON group, the AD-CON group demonstrated glucose intolerance, and this was not observed in the AD-ZXT and AD-LTN groups suggesting zeaxanthin and lutein supplementation prevented the development of glucose intolerance. However, AD-RVT did not improve glucose tolerance compared to AD-CON. The area under the curve (AUC) of the 1st and 2nd phases of serum glucose concentrations was higher in the AD-CON than in the Normal-CON group, and this was lower in the AD-ZXT and AD-LTN groups (*p* < 0.01 and *p* < 0.01), indicating the role of zeaxanthin and lutein intake in lowering the AUC (Figure 4B). 

**Figure 5:**
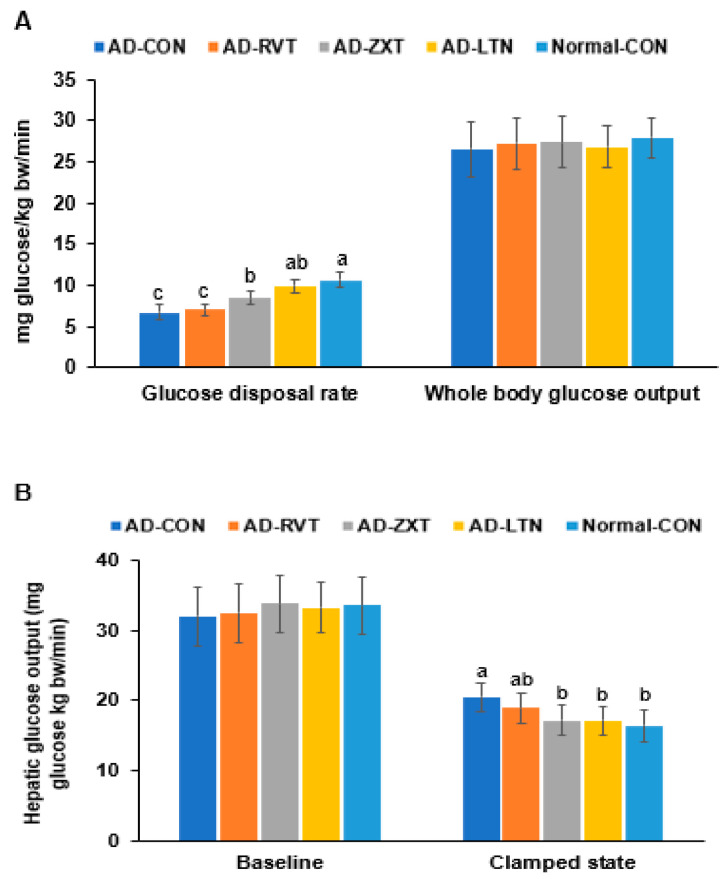
Metabolic parameters during the hyperinsulinemic–euglycemic clamp. (**A**). Whole-body glucose infusion rates (GIR) and glucose uptake. (**B**). Hepatic glucose output at the basal and clamped states (n = 8). Bars and error bars represent mean ± standard deviation. Each dot and bar with error bars represent mean ± SD (n = 8 for each group). The β-amyloid (25-35)-infused rats were fed high-fat diets with 200 mg/kg bw resistant dextrin (AD-CON; control), lutein (AD-LTN), zeaxanthin (AD-ZXT), or resveratrol (AD-RVT; positive-control). The rats infused with β-amyloid (35-25) were fed a high-fat diet containing 200 mg resistant dextrin per kg bw and served as normal controls (Normal-CON, Non-AD). Different letters (a, b, c) on the bars are significantly different from each other (*p* < 0.05) by Tukey’s post hoc test. Bars with the same letter (a, a) are not significantly different.

In the hyperinsulinemic–euglycemic clamp, the whole-body glucose disposal rate was much lower in the AD-CON than in the Normal-CON group. The rate in the AD-LTN group was as high as in the Normal-CON group (*p* < 0.001). However, the whole-body glucose uptake was not significantly different among the groups (Figure 5A). The hepatic glucose output at baseline, representing fasting serum glucose concentrations, did not differ. However, the hepatic glucose output was higher in the AD-CON group than in the Normal-CON group. The hepatic glucose output in the AD-ZXT and AD-LTN groups, but not AD-RVT, was similar to the Normal-CON group (*p* < 0.05; Figure 5B), suggesting that zeaxanthin and lutein regulated the output to prevent the increase when serum glucose concentrations were maintained at euglycemia by injecting insulin. Therefore, the AD-CON group showed insulin resistance, wherein the glucose disposal rate decreased, and hepatic glucose output increased during hyperinsulinemia. The intake of zeaxanthin and lutein suppressed the increase in insulin resistance observed in AD, while resveratrol minimally decreased it. 

**Figure 6:**
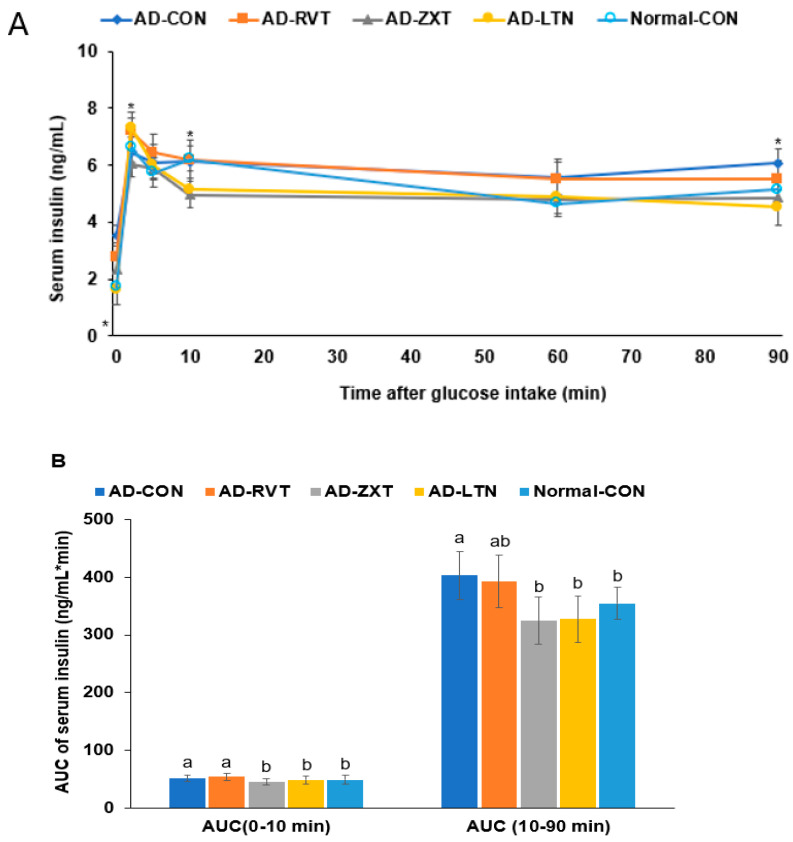
Insulin secretion capacity during the hyperglycemic clamp. (**A**). Changes in the serum insulin concentrations during the hyperglycemic clamps (n = 8 for each group). Serum insulin levels were measured when serum glucose levels were maintained at 5.5 mM above fasting levels. (**B**). Area under the curve (AUC) of serum insulin concentration. Each dot and bar with error bars represent mean ± SD (n = 8). The β-amyloid (25-35)-infused rats were fed high-fat diets with 200 mg/kg bw resistant dextrin (AD-CON; control), lutein (AD-LTN), zeaxanthin (AD-ZXT), or resveratrol (AD-RVT; positive-control). The rats infused with β-amyloid (35-25) were fed a high-fat diet containing 200 mg resistant dextrin per kg bw and served as normal controls (Normal-CON, Non-AD). Dots and error bars represent mean ± standard deviation. * Significantly different at the time point among the four groups at *p* < 0.05 by one-way ANOVA. Different letters (a, b) on the bars are significantly different from each other (*p* < 0.05) by Tukey’s post hoc test. Bars with the same letter (a, a) are not significantly different.

In the hyperglycemic clamp, by infusing glucose to maintain serum glucose concentrations at 220 mg/dL, the serum insulin levels at 2 min reached the peak. Then, they decreased until 10 min, called the acute phase insulin secretion. They were continuously increased or maintained until the clamp was finished, called the second phase insulin secretion (Figure 6A). The serum insulin concentrations at 2 min were lower in the AD-CON than in the Normal-CON group. It increased in the AD-ZXT and AD-LTN groups but not in the AD-RVT group (*p* < 0.05; Figure 6A, Table 2). The serum insulin levels at 10 min were lower in the AD-ZXT and AD-LTN groups than in the other groups. In the second phase of insulin secretion, the serum insulin concentrations were higher in the AD-CON group than in the Normal-CON group, and they decreased in the AD-ZXT and AD-LTN groups but not AD-RVT, as much as in the Normal-CON group (*p* < 0.05; Table 2). The AUC of the acute and second phases was higher in the AD-CON group than in the Normal-CON group and lower in the AD-ZXT and AD-LTN groups (*p* < 0.05; Figure 6B). These results suggest that AD impaired the acute phase insulin secretion and pattern compared to the Normal-CON group. AD-ZXT and AD-LTN, but not AD-RVT, improved the impairment of insulin secretion compared to the AD-CON group, suggesting zeaxanthin and lutein prevented abnormal insulin secretion. 

## 3. Discussion 

The present study investigated the efficacy and explored the mechanisms of the carotenoids, zeaxanthin, and lutein, and the polyphenol resveratrol in modulating an AD-like pathology, insulin resistance, and insulin secretion using an Aβ(25-35) AD rat model. The results demonstrated that zeaxanthin and lutein effectively attenuated memory impairment and other AD-like pathologies in Aβ(25-35) rats by potentially modulating neuroinflammation, hippocampal and systemic insulin resistance, and AChE activity. 

The development of AD has been linked to increased AChE activity and impaired insulin/insulin-like growth factor-1(IGF-1) signaling in the hippocampus [17]. In the current study, the AD rat model exhibited Aβ deposition, attenuated hippocampal insulin signaling (pAkt→GSK-3β), and increased neuroinflammation and AChE activity. As measured by behavioral tests, the changes contribute to regulating neuronal function and plasticity to develop memory deficits. These findings in this animal model are consistent with established AD-like pathologies [18]. 

The findings from this study also highlight the vital link between systemic insulin resistance and the pathology of Alzheimer’s disease. In addition to the impaired hippocampal insulin signaling observed in the AD rat model, they also exhibited whole-body insulin resistance and high hepatic glucose output. Interestingly, the degree of memory impairment correlated with systemic and hepatic insulin resistance [19]. Increasing evidence from epidemiological and mechanistic studies supports this relationship between insulin resistance and AD pathology [20,21]. Some research studies have suggested that AD could also be classified as a type of diabetes and called type 3 diabetes because individuals with chronically high blood sugar often develop late-onset AD. Insulin resistance, a key feature of type 2 diabetes and metabolic syndrome, has been identified as a risk factor for the development of AD. Systemic insulin resistance is positively correlated with tau and Aβ deposition [21]. Impairments in brain insulin signaling and glucose tolerance are believed to contribute to the neurodegeneration and cognitive deficits seen in AD. 

The brain is considered an insulin-insensitive organ. Insulin receptors are present throughout the brain and serve essential functions in whole-body metabolism and brain function. Insulin regulates neurotransmitter release, neuronal survival, synaptic plasticity, and memory formation [22]. Disruptions in the insulin signaling pathways can lead to the dysregulation of these processes. Additionally, insulin resistance can exacerbate AD pathologies, such as Aβ and tau protein accumulation, neuroinflammation, and oxidative stress [22]. This study showed that Aβ infusion to the hippocampus attenuated insulin signaling in the hippocampus, increased neuroinflammation, and exacerbated systemic glucose intolerance and insulin resistance, resulting in an AD-like pathology in the animal model.

Notably, our results showed that zeaxanthin and lutein supplementation effectively inhibited impaired glucose disposal and elevated hepatic glucose output in the AD rat model, restoring insulin sensitivity to levels similar to non-AD rats. It suggests that carotenoids may attenuate AD pathology, at least partially, by modulating systemic insulin resistance and consequent disruptions in brain insulin/glucose metabolism. Previous randomized clinical trials have demonstrated that lutein/zeaxanthin supplementation (20 mg/day for 6 months) improves insulin sensitivity and reduces inflammation compared to a placebo in overweight/obese individuals [23]. Lutein or zeaxanthin supplementation ameliorates insulin resistance and glucose tolerance and reduces oxidative stress in obese rats fed a high-fat diet [24]. Furthermore, an observational study has found that higher serum levels of zeaxanthin and lutein were associated with better cognitive performance and a reduced risk of cognitive impairment in older adults [25]. Studies have also shown that zeaxanthin and lutein supplementations improve memory function in mice animal models [13]. There are several potential mechanisms by which zeaxanthin and lutein may attenuate AD pathology and alleviate memory function through the modulation of insulin resistance and brain insulin signaling. In the current study, zeaxanthin and lutein effectively inhibited systemic insulin resistance, as evidenced by improved glucose disposal and reduced hepatic glucose output in the AD rat model. This improvement in peripheral insulin sensitivity could indirectly benefit brain function by preventing the detrimental effects of hyperglycemia, dyslipidemia, and other metabolic disturbances associated with insulin resistance [26]. Improved metabolic control may reduce oxidative stress, inflammation, and vascular dysfunction, contributing to neurodegeneration and cognitive impairment. The anti-inflammatory and antioxidant properties of zeaxanthin and lutein may also benefit brain insulin signaling and cognitive function. Chronic inflammation and oxidative stress impair the insulin signaling pathways and exacerbate neurodegeneration [27]. By suppressing neuroinflammation (reduction in *TNF-α*, *IL-1β*) and lipid peroxidation, these carotenoids may create a more favorable environment for optimal insulin sensitivity and neuronal function in the hippocampus. 

Lutein and zeaxanthin demonstrated improved memory function through enhancing systemic and brain insulin sensitivity and reducing neuroinflammation in our study. These effects are likely due to direct action in the brain, as both compounds can traverse the blood–brain barrier (BBB) [28]. The presence of these carotenoids in human brain tissue, with lutein showing preferential accumulation, has significant implications for brain physiology [29]. Their BBB permeability allows direct interaction with neural tissues, potentially influencing antioxidant status, inflammatory processes, and cellular signaling pathways in the brain. However, the specific distribution patterns in different brain regions and the concentrations achieved in brain tissue following supplementation require further investigation.

Resveratrol supplementation has been reported to improve learning and memory with decreased Aβ deposition, mitigated neuronal loss, enhanced hippocampal neurogenesis, and improved synaptic plasticity in animal studies [15,30]. A period of 14–26 weeks of resveratrol supplementation has been demonstrated to improve memory performance and functional connectivity in the hippocampus of older people and post-menopausal women [31,32]. This study used resveratrol supplementation as a positive control. This study also showed that resveratrol supplementation improved memory function by enhancing hippocampal insulin signaling and systemic insulin sensitivity in Aβ-infused rats. However, it did not suppress AChE activity. Lutein and zeaxanthin supplementation was superior to resveratrol in improving memory function and inhibiting AChE activity. 

This study examined the effects of lutein and zeaxanthin separately to isolate their impacts on AD pathology and cognitive function. It allowed us to delineate the specific contributions of each compound to understand their action mechanisms. However, we acknowledge that this design does not capture potential synergistic effects that might occur when these compounds are used in combination, as they often appear together in natural sources. Future studies should investigate the potential synergistic effects of lutein and zeaxanthin when administered together, as their combined effects could potentially exceed those observed with individual treatments.

In conclusion, AD increases both central and systemic insulin resistance compared to the normal control. It suggested that maintaining glucose homeostasis could be pivotal in preventing and ameliorating AD risk. Zeaxanthin and lutein supplementation attenuated AD-like pathologies by potentially modulating neuroinflammation, hippocampal and systemic insulin resistance, and AChE activity. Their efficacy was better than resveratrol in the AD model. It suggests that the intake of zeaxanthin and lutein has practical implications in preventing or slowing down AD progression in humans. However, further clinical trials are needed to evaluate their efficacy and safety for therapeutic use. 

## 4. Materials and Methods

### 4.1. Animals and Diets 

Male Sprague Dawley (SD) rats, initially weighing 192 ± 30 g, were individually housed in stainless steel cages under controlled conditions (23 °C, 12 h light/dark cycle). All procedures adhered to guidelines approved by the Animal Care and Use Review Committee at Hoseo University, Korea (HSIACUC-19-011). Following a 7-day acclimation period, rats were anesthetized with an intraperitoneal injection of ketamine and xylazine (100 mg and 10 mg/kg body weight, respectively) for stereotaxic surgery. A stainless-steel cannula was implanted to connect an osmotic pump to the bilateral CA1 subregions of the hippocampus (coordinates: lateral, −3.3 mm from bregma; posterior, 2.0 mm from midline; ventral, −2.5 mm from dura). For AD induction, β-amyloid (25-35) from Sigma Co., St. Louis, MO, USA) was infused at 3.6 nmol/day for 14 days, which induced neuropathological features of AD, including Aβ deposition, neuroinflammation, oxidative stress, insulin resistance, and cognitive deficits, providing a valuable model to investigate potential therapeutics [5,12]. Non-AD rats received β-amyloid (35-25), having a reverse sequence of β-amyloid (25-35), at the same rate, which does not accumulate in the brain. 

**Figure 7:**
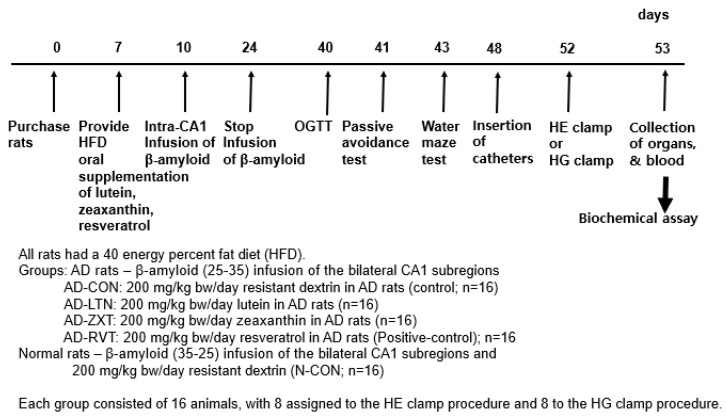
Experimental design. HE clamp, hyperinsulinemic–euglycemic clamp; HG clamp, hyperglycemic clamp.

The AD rats infused with β-amyloid (25-35) were randomly assigned to four groups (n = 16 per group): AD-CON (control), AD-LTN, AD-ZXT, and AD-RVT (positive-control). Each group received a daily oral gavage of either 200 mg resistant dextrin per kg body weight (placebo; Samyang, Seongnam, Korea), lutein, zeaxanthin, or resveratrol, respectively (Figure 7). The lutein (95% purity), zeaxanthin (95% purity), and resveratrol (99% purity) were sourced from Green Spring (Xi’an, China). Rats infused with β-amyloid (35-25) received 200 mg/kg body weight resistant dextrin daily and served as the normal control (Normal-CON, Non-AD; n = 16). Resistant dextrin was selected as the placebo for AD-CON and Normal-CON due to its non-caloric nature, which matches the low caloric content of lutein and zeaxanthin. Additionally, resistant dextrin does not have a function to interfere with AD function, minimizing potential confounding physiological effects. This choice helps to ensure that any observed effects in the treatment groups could be attributed to the active compounds in lutein, zeaxanthin, and resveratrol. 

While traditional AD interventions like AChE inhibitors (e.g., donepezil) are effective and widely used clinically [33], they were not appropriate for the Aβ-infused rat model. Our choice of resveratrol as a positive control (AD-RVT group) was based on its particular relevance to the Aβ-infused rat model and the study on natural compounds. Resveratrol has well-documented neuroprotective properties and has shown specific benefits in Aβ-related AD pathology [34]. Our focus was on comparing natural compounds with similar antioxidant and anti-inflammatory properties, and resveratrol’s efficacy in Aβ models made it an appropriate benchmark for our experimental compounds.

Throughout the 8-week intervention period, all rats including AD rats and non-AD rats had ad libitum access to water and a high-fat diet, along with their assigned oral supplement. A high-fat diet (consisting of 40% energy from fat) was chosen to reflect the increasing worldwide trend in elevated dietary fat intake. Previous research has shown that a high-fat diet can exacerbate Aβ-related pathology and cognitive deficits in animal models of AD [5,12]. We investigated how this dietary factor may interact with and accelerate AD development, while also allowing for a direct comparison of its effects on normal brain function versus AD-related pathology. 

The high-fat diet was based on the AIN-93 diet formulation but modified to be high in fat. The diet composition was as follows: 40% fat, 20% protein, and 40% carbohydrates, as % of total energy. The primary sources were lard (CJ Co., Seoul, Republic of Korea) for fat, casein for protein, and a combination of starch and sugar for carbohydrates. Food intake was measured daily, and body weights were recorded weekly to adjust supplement doses. After overnight fasting, serum glucose and insulin levels were measured weekly.

### 4.2. Passive Avoidance Test 

On day 41 of this study, the rats were tested for short-term memory deficits using a passive avoidance apparatus consisting of a two-compartment dark/light shuttle box [5]. In the acquisition trial, electric stimulation (75 V, 0.2 mA, 50 Hz) was delivered for 5 s immediately after the rats had entered the dark chamber. Five seconds later, the rats were removed from the dark chamber and returned to their home cage. After 24 h, the retention latency time was measured similarly to that in the acquisition trial. However, electric foot stimulation was not delivered, and the latency time was recorded to a maximum of 600 s. Short latencies indicate a memory deficit, compared to significantly longer latencies.

### 4.3. Water Maze Test 

As previously described [5], spatial memory function was assessed on day 43 with a Morris water maze test. The Morris water maze tests examined hippocampal-dependent learning, especially long-term spatial memory. It was conducted in a circular pool with a diameter of 180 cm and a height of 50 cm. The pool was filled with water to a depth of 35 cm, maintained at a temperature of 23 ± 1 °C. Black edible dye was added to the water to create a dark background, enhancing the visibility of the rats. The pool was divided into four quadrants, and a circular platform, 10 cm in diameter and 30 cm in height was placed in a fixed location. The platform was submerged so that its top was 2 cm below the water’s surface, making it invisible to the rats. The water maze test consisted of 2 training trials per day, with the platform remaining in the same position across trials. Each rat was placed individually in the pool, facing the wall, to minimize directional bias at the starting point. During each trial, the rat was given a maximum of 8 min to locate the platform. If the rat failed to find the platform within the allotted time, the researcher gently guided the rat to the platform, where it remained for 30 s to reinforce learning. On the fourth day, a probe trial was conducted without the platform in the pool to assess memory retention. The free movement of the rat in each trial was recorded by a video tracking system (Ethovision system; Noldus, Wageningen, The Netherlands). The time and distance it took to reach the area where the platform had been located were measured during the test trial.

### 4.4. Oral Glucose Tolerance 

Overnight fasted rats had 2 g glucose per kg body weight orally, and serum glucose concentrations were measured every 10 min until 90 min, and then lastly at 120 min. 

### 4.5. Hyperinsulinemic–Euglycemic Clamp to Assess Insulin Sensitivity/Resistance 

On day 48, all rats in each group (n = 16) underwent catheterization of the right carotid artery and left jugular vein. At 5–6 days after the catheter implantation, a hyperinsulinemic–euglycemic clamp was performed on 8 fasted conscious rats randomly selected from 16 rats of each group to determine insulin resistance, as previously described [35,36]. [3-^3^H] glucose (Perkin Elmer, Wellesley, MA, USA) was continuously infused for four hours at the rate of 0.05 μCi/min. The basal hepatic glucose output was measured in blood collected at 100 and 120 min after initiating the (3-^3^H) glucose infusion. A primed continuous infusion of human regular insulin (Humulin; Eli Lilly, Indianapolis, IN) was then initiated at 20 pmol/kg bw/min to raise the plasma insulin concentrations to approximately 1100 pM after 210–240 min. Blood samples were collected at 10 min intervals, and 25% glucose was infused as needed to clamp glucose levels at approximately 6 mM. The whole-body glucose uptake and basal glucose turnover rates were determined according to the ratio of the [3-^3^H] glucose infusion rate to the specific activity of plasma glucose (dpm/mmol) during the final 30 min. Hepatic glucose production in the hyperinsulinemic clamped state was determined by subtracting the glucose infusion rate from the whole-body glucose uptake. 

### 4.6. Hyperglycemic Clamp to Determine the Insulin Secretory Capacity 

At 5–6 days after implantation, a hyperglycemic clamp to determine insulin secretory capacity was performed in the remaining eight rats in each group under free-moving and overnight fasted conditions. During the clamp, a glucose infusion maintained serum glucose levels at 5.5 mM above the baseline, and serum glucose and insulin levels were measured at 0, 2, 5, 10, 60, 90, and 120 min [36]. The serum glucose concentrations were measured with a Beckman glucose analyzer, and serum insulin concentrations were measured with an Ultrasensitive insulin ELISA kit (Crystal Chem, Elk Grove Village, IL, USA). 

Two days after the clamp, eight randomly selected rats from each group were injected with bromodeoxyuridine (BrdU, 100 µg/kg body weight). Six hours post-injection, the rats were anesthetized with intraperitoneal injections of a mixture of ketamine and xylazine and injected with regular human insulin (5 U/kg body weight) through the inferior vena cava. Ten minutes later, they were sacrificed by decapitation; liver and brain tissues were collected and frozen in liquid nitrogen and stored at –70 °C. Half of the brain was used for immunohistochemistry, and the hippocampus was dissected from the other half of the brain and divided into two portions. One portion was used to measure RNA extract, and the other was used for the other assays. Liver and hippocampal glycogen content was determined after centrifuging the lysates at 3000 rpm for 10 min and deproteinizing the supernatant using 1.5N perchloric acid. The supernatants were digested with α-amyloglucosidase, and the glucose content was measured using a glucose spectrophotometric kit. The glycogen content was calculated from glucose released from the glycogen [5,35]. Triglycerides were extracted with chloroform/methanol (2:1, vol/vol) from the brain resuspended in pure chloroform and determined as previously described [5] using a Trinder kit (Asan Pharm., Daejeon, Korea). AChE activity in the hippocampal extract was assessed using an AChE enzyme-linked immunosorbent assay (ELISA) kit (DoGenBio, Ansan, Korea). The serum levels of tumor necrosis factor-α (*TNF-α*) and interleukin (IL)-1β were measured using the respective ELISA kits (Thermo Fisher Scientific, Waltham, MA, USA).

### 4.7. Brain Immunohistochemistry to Evaluate Amyloid-β Deposition

The brain tissues retained for immunohistochemistry were immediately dissected from the body, immersed in 20% sucrose solution, and stored in −20 °C freezer [5,12]. Cryoprotected frozen brain tissues were serially sectioned on a cryostat (Leica, Wetzlar, Germany) into 30 μm coronal sections, and β-amyloid accumulation in the hippocampus was determined by immunohistochemistry using β-amyloid antibodies. The β-amyloid deposition was calculated as the % β-amyloid-positive cells in the hippocampus area. 

### 4.8. Quantitative Realtime PCR and Immunoblotting

The hippocampal tissues were collected from three randomly selected rats per group. They were divided into two portions, and one portion was used to isolate total RNA using a monophasic solution of phenol and guanidine isothiocyanate (Trizol reagent, Invitrogen, Rockville, MD, USA). The cDNA was produced using a mixture of total RNA, superscript III reverse transcriptase, and high-fidelity Taq DNA polymerase (1:1:1, v:v:v) by polymerase chain reaction (PCR) and mixed with the primers of the genes of interest and SYBR Green mix. The expressions of genes of interest were determined using a realtime PCR machine (BioRad Laboratories, Hercules, CA, USA). The primers used for ciliary neurotrophic factor (CNTF), brain-derived neurotrophic factor (*BDNF*), *TNF-α*, *IL-1β*, and β-actin were as previously described [5]. Gene expression levels in samples were quantitated using the comparative cycle of threshold (CT) method [5,37]. 

The other portion of the hippocampus was used for immunoblotting. Each tissue was lysed with a radioimmunoprecipitation assay buffer (RIPA) lysis buffer containing protease inhibitors, and the lysate protein contents were measured using a Bio-Rad protein assay kit (Hercules, CA, USA). Lysates from two rats were used for immunoblotting, which was performed in two sets. The lysates with proteins (30–50 μg) were resolved into sodium dodecyl sulfate-polyacrylamide gel electrophoresis (SDS PAGE), and the amount of the proteins of interest was examined with the specific antibodies as follows: protein kinase B (PKB or Akt), phosphorylated PKB^Ser473^, glycogen synthase kinase (GSK)-3β, phosphorylated GSK-3β^ser9^, phosphorylated tau^ser396^ and tau (Cell Signaling Technology, Danvers, MA, USA), and β-actin (Santa Cruz Biotech, Dallas, TX, USA). The intensity of the proteins of interest was measured using Imagequant TL (Amersham Biosciences, Piscataway, NJ, USA). 

### 4.9. Statistical Analysis

The statistical analysis was conducted using SAS version 7 (SAS Institute in Cary, NC, USA). It was determined that a sample size of 8 rats per group would offer adequate power to evaluate the main effects, calculated using the G power program with a power of 0.85 and an effect size of 0.50. A hyperglycemic clamp and euglycemic hyperinsulinemic clamp cannot be performed in one rat, and 16 rats were used for each group. The results are expressed as means ± standard deviations (SDs). Univariate analysis (Proc Univariate) was used to test whether the results showed a normal distribution. If the data were normally distributed, a one-way analysis of variance (ANOVA) was utilized to compare the statistical differences among the groups. Tukey’s test was applied for multiple group comparisons in cases where the one-way ANOVA revealed a significant difference between the groups. If the data did not have a normal distribution, the Kruskal–Wallis H test was applied to analyze the statistical difference among the groups. Statistical significance was defined as *p* < 0.05.

## Figures and Tables

**Table 1:** Hippocampal contents related to Alzheimer’s disease.

	AD-CON	AD-RVT	AD-ZXT	AD-LTN	Normal-CON
Triglyceride (ug/mg protein)	19.4 ± 2.0 ^a^	17.0 ± 1.6 ^b^	20.4 ± 1.7 ^a^	17.2 ± 1.9 ^b^	17.3 ± 1.8 ^b^
Cholesterol (mg/dL)	5.21 ± 0.47 ^a^	4.20 ± 0.48 ^b^	5.35 ± 0.49 ^a^	4.51 ± 0.51 ^b^	4.60 ± 0.43 ^b^
Glycogen (mg/dL)	3.39 ± 0.47 ^c^	4.44 ± 0.46 ^ab^	4.28 ± 0.47 ^b^	4.31 ± 0.41 ^b^	4.94 ± 0.53 ^a^
Lipid peroxides (MDA μmol/g tissue)	0.78 ± 0.08 ^a^	0.67 ± 0.08 ^b^	0.57 ± 0.07 ^c^	0.54 ± 0.07 ^c^	0.51 ± 0.06 ^c^
Acetylcholinesterase (U/mg protein)	0.34 ± 0.05 ^a^	0.31 ± 0.05 ^a^	0.22 ± 0.04 ^b^	0.21 ± 0.04 ^b^	0.14 ± 0.04 ^c^
TNF-α content (pg/mg protein)	12.5 ± 0.93 ^a^	10.9 ± 0.84 ^b^	10.4 ± 0.88 ^bc^	9.72 ± 0.73 ^c^	10.1 ± 0.84 ^bc^
*IL-1β* content (pg/mg protein)	11.6 ± 0.98 ^a^	10.4 ± 0.94 ^b^	9.84 ± 0.89 ^b^	9.31 ± 0.82 ^bc^	8.93 ± 0.79 ^c^
mRNA *TNF-α* (AU)	1.0 ± 0.0 ^a^	0.82 ± 0.09 ^b^	0.75 ± 0.09 ^bc^	0.71 ± 0.08 ^c^	0.73 ± 0.09 ^c^
mRNA *IL-1β* (AU)	1.0 ± 0.0 ^a^	0.86 ± 0.09 ^b^	0.73 ± 0.07 ^c^	0.79 ± 0.08 ^bc^	0.71 ± 0.08 ^c^
mRNA *BDNF* (AU)	1.0 ± 0.0 ^c^	1.61 ± 0.22 ^b^	1.86 ± 0.17 ^b^	2.44 ± 0.21 ^a^	2.51 ± 0.07 ^a^
mRNA *CTNF* (AU)	1.0 ± 0.0 ^c^	1.93 ± 0.27 ^b^	2.27 ± 0.27 ^ab^	2.51 ± 0.26 ^a^	2.60 ± 0.25 ^a^

Values are mean ± SD (n = 16 for each group). The β-amyloid (25-35)-infused rats fed high-fat diets with 200 mg/kg bw/day resistant dextrin (AD-CON; control), lutein (AD-LTN), zeaxanthin (AD-ZXT), or resveratrol (AD-RVT; positive-control). The rats infused with β-amyloid (35-25) had a high-fat diet containing 200 mg/kg bw/day resistant dextrin as a normal control (Normal-CON). MDA, malondialdehyde; TNF, tumor necrosis factor; IL, interleukin; *BDNF*, brain-derived neurotrophic factor; *CTNF*, ciliary neurotrophic factor; AU, arbitrary unit. Values with different superscript letters (a, b, c) are significantly different from each other (*p* < 0.05) by Tukey’s post hoc test. Values with the same superscript letter (a, a) are not significantly different.

**Table 2:** Energy, glucose, and lipid metabolism and liver damage index.

	AD-CON	AD-RVT	AD-ZXT	AD-LTN	Normal-CON
Final body weight (g)	412 ± 10.9 ^bc^	404 ± 16.5 ^c^	422 ± 16.2 ^ab^	424 ± 19.4 ^ab^	439 ± 13.3 ^a^
Weight gain (g)	207 ± 6.02 ^c^	199 ± 7.13 ^c^	217 ± 8.42 ^b^	220 ± 9.9 ^b^	237 ± 7.23 ^a^
Visceral fat (g)	5.43 ± 0.98	4.74 ± 0.88	5.04 ± 0.86	5.57 ± 0.81	5.45 ± 0.80
Serum *TNF-α*	59.5 ± 5.21 ^a^	52.3 ± 4.67 ^b^	52.7 ± 4.57 ^b^	47.3 ± 4.36 ^c^	49.7 ± 4.67 ^bc^
Serum glucose (mg/dL)	121 ± 6.52	117 ± 4.47	114 ± 4.23	116 ± 4.83	116 ± 3.52
Fasting serum insulin (ng/mL)	3.54 ± 0.37 ^a^	2.76 ± 0.53 ^b^	2.32 ± 0.52 ^b^	1.63 ± 0.54 ^c^	1.71 ± 0.58 ^c^
HOMA-IR	14.2 ± 1.13 ^a^	10.7 ± 1.23 ^b^	8.84 ± 1.17 ^c^	6.30 ± 1.17 ^d^	6.59 ± 1.23 ^d^
Serum insulin at 2 min (ng/mL)	6.45 ± 0.66 ^c^	7.18 ± 0.67 ^a^	6.05 ± 0.56 ^c^	7.32 ± 0.32 ^a^	7.13 ± 0.48 ^b^
Serum insulin at 10 min	6.12 ± 0.58 ^a^	6.17 ± 0.62 ^a^	4.97 ± 0.45 ^b^	5.15 ± 0.63 ^b^	6.21 ± 0.66 ^a^
Serum insulin at 60 and 90 min	5.82 ± 0.51 ^a^	5.50 ± 0.66 ^a^	4.82 ± 0.66 ^b^	4.70 ± 0.65 ^b^	4.90 ± 0.41 ^b^
Serum total cholesterol	142 ± 12.7 ^a^	146 ± 13.2 ^a^	112 ± 12.1 ^b^	143 ± 14.3 ^a^	138 ± 11.6 ^a^
Serum HDL	26.5 ± 3.4 ^b^	34.1 ± 3.7 ^a^	25.1 ± 2.9 ^b^	31.6 ± 3.8 ^a^	32.4 ± 3.3 ^a^
Serum TG	91.8 ± 8.4 ^a^	93.3 ± 7.8 ^a^	80.1 ± 8.2 ^b^	77.6 ± 7.9 ^b^	72.6 ± 6.7 ^b^
Serum ALT	54.3 ± 5.1 ^a^	53.1 ± 4.8 ^a^	46.3 ± 4.1 ^b^	49.7 ± 5.3 ^ab^	49.9 ± 4.7 ^ab^
Serum AST	57.4 ± 4.9 ^a^	55.9 ± 5.3 ^a^	44.9 ± 4.6 ^c^	41.5 ± 3.8 ^c^	51.8 ± 4.7 ^b^

Values are mean ± SD (n = 16 for each group). The β-amyloid (25-35)-infused diabetic rats fed high-fat diets with 200 mg/kg bw resistant dextrin (AD-CON; control), lutein (AD-LTN), zeaxanthin (AD-ZXT), or resveratrol (AD-RVT; positive-control). The rats infused with β-amyloid (35-25) had a high-fat diet containing 200 mg resistant dextrin per kg bw as the normal control (Normal-CON). Values with different superscript letters (a, b, c, d) are significantly different from each other (*p* < 0.05) by Tukey’s post hoc test. Values with the same superscript letter (a, a) are not significantly different. TNF, tumor necrosis factor; HOMA-IR, homeostatic model assessment for insulin resistance; HDL, high-density lipoprotein; TG, triglyceride; ALT, alanine aminotransferase; AST, aspartate aminotransferase.

## Data Availability

All data are included in the manuscript.
